# Elevated particulate organic carbon export flux induced by internal waves in the oligotrophic northern South China Sea

**DOI:** 10.1038/s41598-018-20184-9

**Published:** 2018-02-01

**Authors:** Dewang Li, Wen-Chen Chou, Yung-Yen Shih, Guan-Yu Chen, Yi Chang, Chun Hoe Chow, Tsang-Yuh Lin, Chin-Chang Hung

**Affiliations:** 1 Department of Oceanography, National Sun Yat-sen University, Kaohsiung, 804 Taiwan; 2 Key Laboratory of Marine Ecosystem and Biogeochemistry, Second Institute of Oceanography, State Oceanic Administration, Hangzhou, 310012 PR China; 3 Institute of Marine Environment and Ecology, National Taiwan Ocean University, Keelung, 202 Taiwan; 4 Department of Applied Science, R.O.C Naval Academy, Kaohsiung, 813 Taiwan; 5 Institute of Ocean Technology and Marine Affair, National Cheng Kung University, Tainan, 701 Taiwan; 6 Department of Marine Environmental Informatics, National Taiwan Ocean University, Keelung, 202 Taiwan

## Abstract

To understand the biogeochemical response to internal waves in the deep basin of the northern South China Sea (NSCS), particulate organic carbon (POC) export fluxes were quantified for the first time during the passage of large internal waves using drifting sediment traps attached with hydrographic sensors. Results revealed large variations in temperature, nitrate and chlorophyll *a* (Chl *a*) concentrations during and after internal waves, suggesting that cold nutrient-replete waters may be brought to the euphotic zone in the dissipation zone during and after the passage of internal wave packets, resulted in phytoplankton flourished. Most importantly, POC export fluxes (110.9 ± 10.7 mg C m^−2^ d^−1^) were significantly enhanced after internal waves compared to non-internal wave area (32.6–73.0 mg C m^−2^ d^−1^) in the NSCS. Such elevated POC fluxes may be induced by downward flourished biogenic particles, particle aggregation or converged particles from mixed layer triggered by internal waves.

## Introduction

In density-stratified ocean, lake, and atmosphere, distortions of density feel the restoring of gravity and propagating as internal waves. Primarily generated by tide-topography interaction and wind work on surface^1^, internal waves in ocean interior are important in maintaining downward mixing of density^2^, offshore drilling^3^, and also in biogeochemical processes^4–6^.

**Figure 1:**
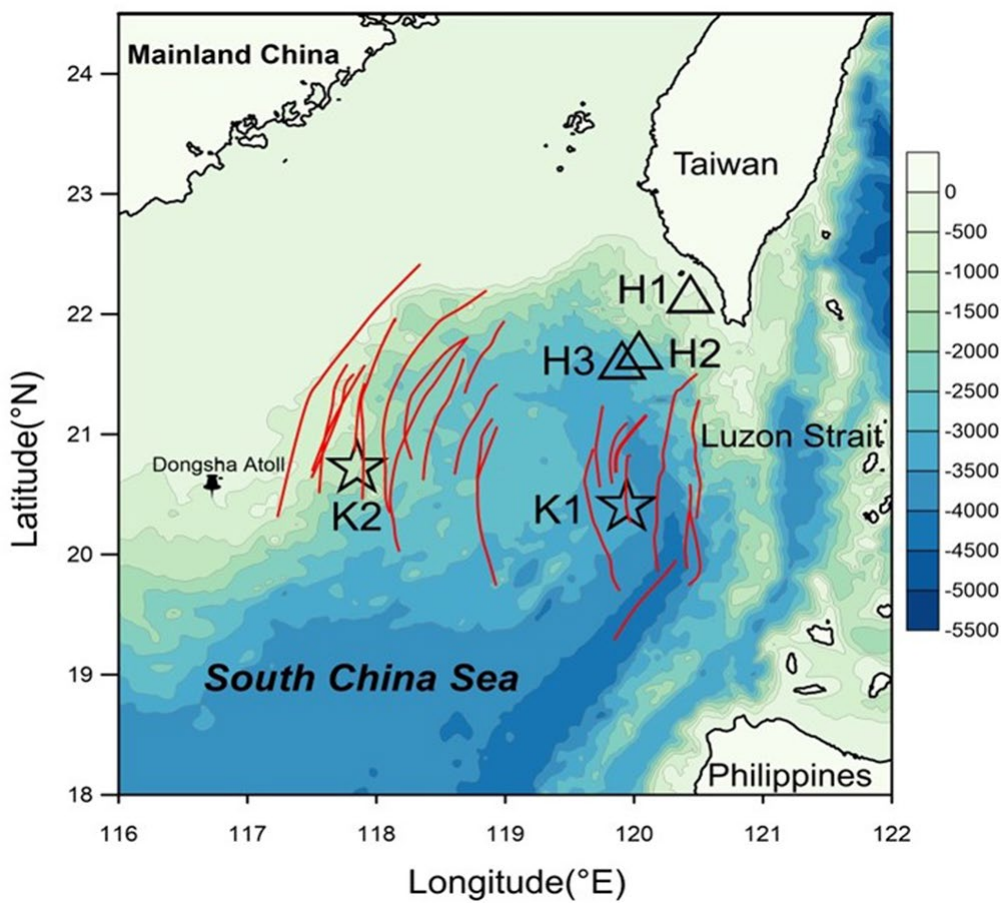
Sampling locations of sediment trap with CTD sensors in the northern South China Sea (The map was created using Surfer software v.12 Surfer, Golden Software). Stations K1 and K2 are denoted by stars. Stations H1, H2, and H3 are denoted by triangles. Black pin shows the position of Dongsha Atoll. Red lines are reported internal waves which were captured by SAR images^7^.

As one of the largest marginal seas in the world, South China Sea (SCS) (Fig. 1) has the largest internal waves reported in the global ocean, revealed by both satellite images^7,8^ and *in situ* observations^9,10^. Originated from Luzon Strait, internal waves propagate westward for thousands of kilometers with amplitudes as high as 150 m^9,10^, and turbulence diffusivity rate is two orders higher than that in open ocean^11^. Internal waves cause upward nutrient flux of 2.4–2.9 mmol N m^−2^ h^−1^ in the northern South China Sea (NSCS)^12^. Enhanced chlorophyll *a* (Chl *a*)^13,14^, and microbial production^15^ resulted by internal waves were observed nearby Dongsha Atoll. However, such satellite-observed Chl *a* increases are likely caused by shifting of subsurface Chl *a* and color dissolved organic matter (CDOM)^16^, thus do not contribute to particulate organic carbon (POC) export. Besides, most of such physical-biogeochemical interaction research focus on the shelf slope area. Little is known about the influence of internal waves on carbon export in the deep basin area of the NSCS, even no POC export data are reported to support the speculation of internal waves’ role in POC export in the deep basin area^17^.

POC export reflects the effect of biological pump by which atmospheric CO_2_ is transferred into ocean as particulate carbon^18,19^, and it supports pelagic ecosystem by providing carbon source^20^. Previous results suggest that the NSCS produces particulate carbon and sinking particulate organic matter in the deep water of the northern South China Sea is predominately marine origin^21,22^. Most research studies do not consider the contributions of episodic events like internal waves which could significantly affect biogeochemical processes and POC export^17^. Similar to typhoons, internal waves were also of short-term scale, thus hard to be captured in traditional cruise survey. In this study, to better understand the effects of internal waves on POC export flux in the deep basin area, we deployed drifting sediment traps and time-series mooring in the propagation route (deep basin area) of internal waves in the NSCS. The POC export flux and possibly mechanism of the observed POC export are presented.

## Results and Discussion

### Hydrographic settings influenced by internal waves

**Figure 2:**
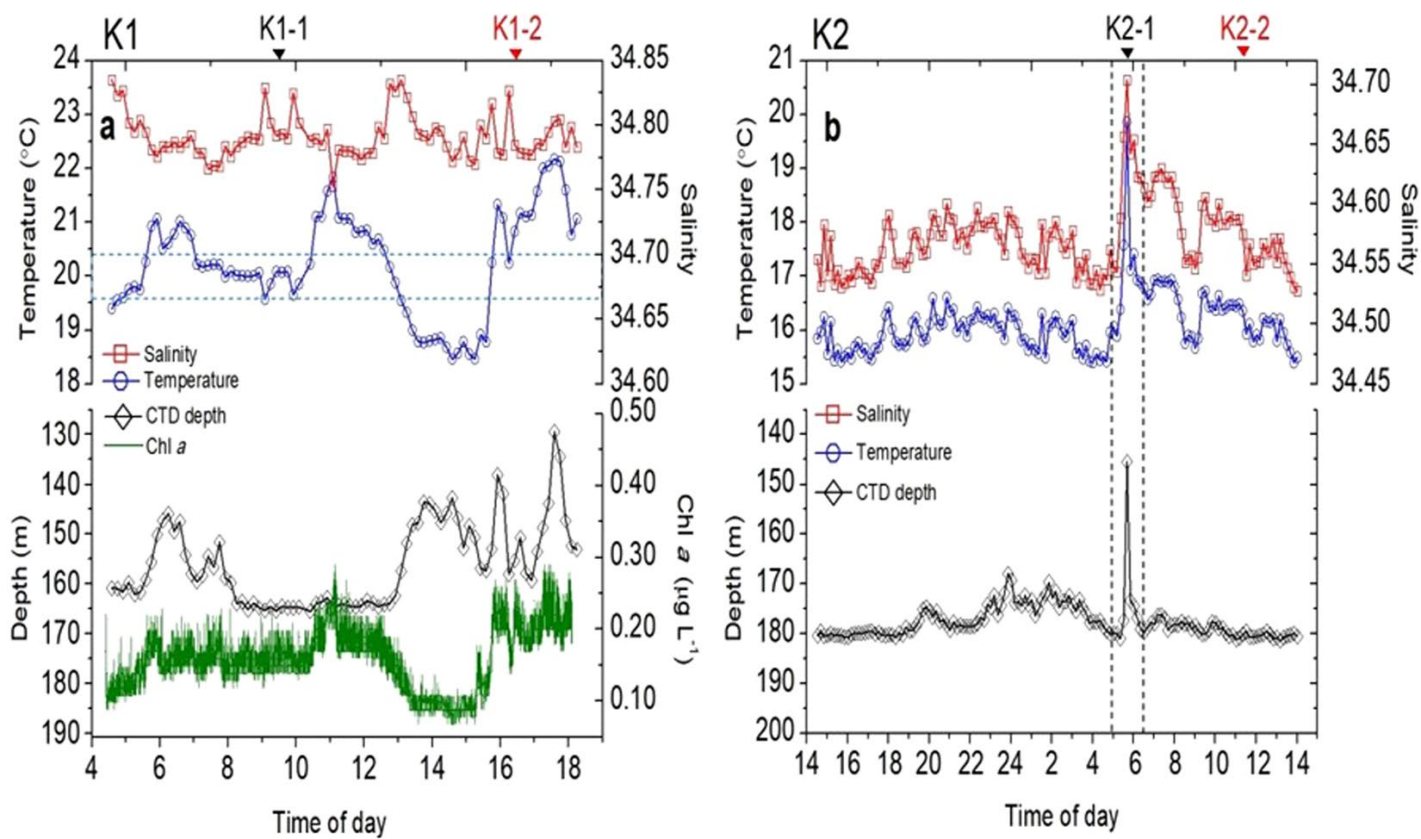
(**a**) Time series variations of temperature, salinity, CTD-sensor depth, and Chl *a* concentration at stations K1 (CTD sensors were deployed at 160 m) and (**b**) K2 (CTD sensors were deployed at 180 m). Light blue dash lines in Fig. 2a show the temperature range of water which is not influenced by internal waves^10^. Note: Chl *a* sensor was not deployed at station K2.

Both deployed depths of CTD sensors, with a stainless frame (>30 kg), varied from 160 m (original deployed depth) to about 130 m at station K1,and from 180 to 145 m at station K2 (Fig. 2) during or after internal waves, respectively. Large variations of time series temperature, salinity, and Chl *a* concentration were observed at stations K1 and K2 (Fig. 2). For example, at station K1, when the CTD sensors were brought to 146 m from 160 m, water temperature increased to 21.0 °C from 19.7 °C during 5~7 am on 6^th^ August (Fig. 2a). Such a short-period (2 hours) variation of temperature, salinity and sensors (including CTD sensors and the frame, ~30 kg) vertical displacement reveals that sediment traps and hydrographic sensors experienced internal waves at station K1. After that, a remarkable warm water signal (21~22 °C) was observed at depth of 160 m during 10~12 am on 6^th^ August, suggesting warm water might be carried down to deep depths. Following the warm shock, the CTD sensors were up and down from 160 to 130 m, and measured large variations of temperature (ranging from ~18 to 22 °C, see Fig. 2a) during 1~6 pm at station K1, suggesting that high turbulent mixing occurred and influenced biological activity. The repeated high vibration of changes in temperature, salinity and sensors vertical displacement (i.e. CTD depth in Fig. 2) during such a short period within 5 hours evidenced that nonlinear internal waves induced turbulent processes at station K1. Namely, the observed hydrographic changes at station K1 should experience the dissipation zone affected by internal waves.

Reversely, hydrographic settings (variations of temperature, salinity, and depth) at station K2 only showed short timescale variations excluding a strong internal wave event occurred at 5:50 am, suggesting that internal waves indeed affected station K2. However, the internal wave event observed at station K2 near Dongsha Atoll was likely a non-linear internal wave because its turbulent processes showed a large pulse of variations in temperature and salinity (Fig. 2, see the time series between the vertical dashed lines) following by immediately decaying within 1 hour to 2 hours. Clearly, the hydrographic settings varied largely at station K2, but they quickly restored to their normal conditions, i.e. likely belonging to the transmission zone affected by internal waves.

**Figure 3:**
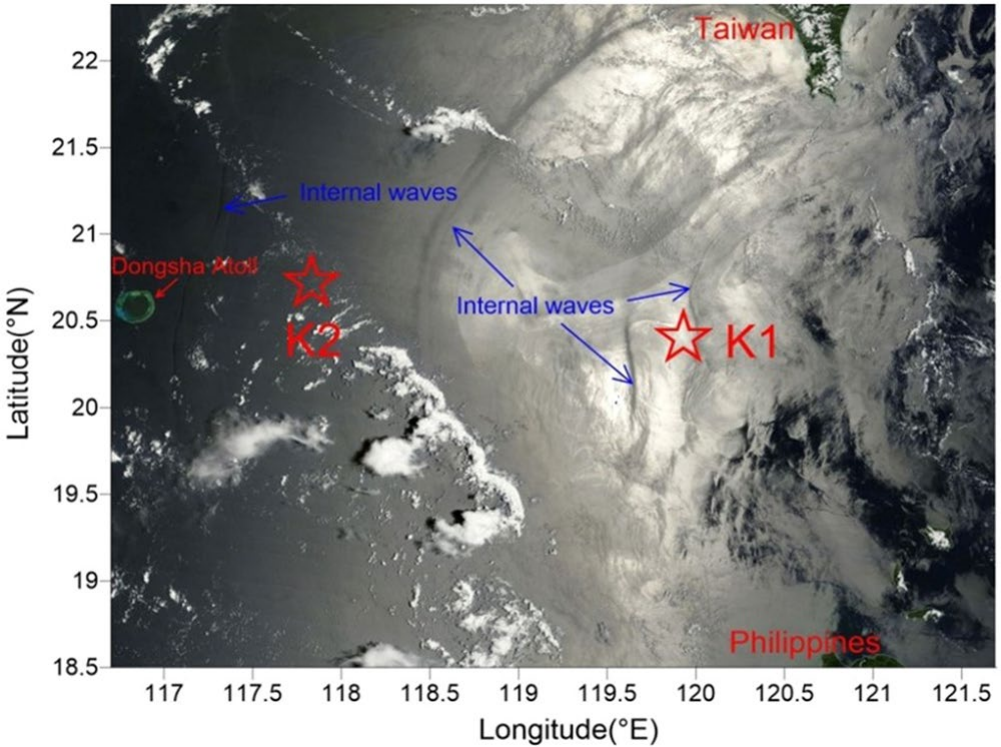
True–color image by Terra MODIS in the South China Sea on 4^th^ August 2016, superimposed by the locations of stations K1 and K2 (red stars). The true-color image in this figure was taken from NASA Worldview (https://worldview.earthdata.nasa.gov/) with an open data policy. This figure was created using Surfer Version 12, Golden Software (http://www.goldensoftware.com/home/terms-of-use).

Similar rapid temperature increase and decrease events in the NSCS were reported by other researchers^14,23^, which can be explained by upward or downward pumping of subsurface layer water. Furthermore, the images of Synthetic-aperture radar (SAR) and Terra MODIS can be used to verify internal wave packets passing stations K1 and K2^7^ (Fig. 1 and Fig. 3). Figure 1 shows the stations K1 and K2 are in the internal-wave active zones observed by SAR. Figure 3 displays a snapshot of Terra MODIS images that observes the internal waves on August 4, 2016, during the experiment at the station K2. Overall, the time series of temperature, salinity, and Chl *a* provide a record of internal waves affecting hydrographic settings in water column at our study area, but the magnitude of such events (and their impact on marine organisms) is difficult to be quantified.

### Changes in water column properties after internal waves

**Figure 4:**
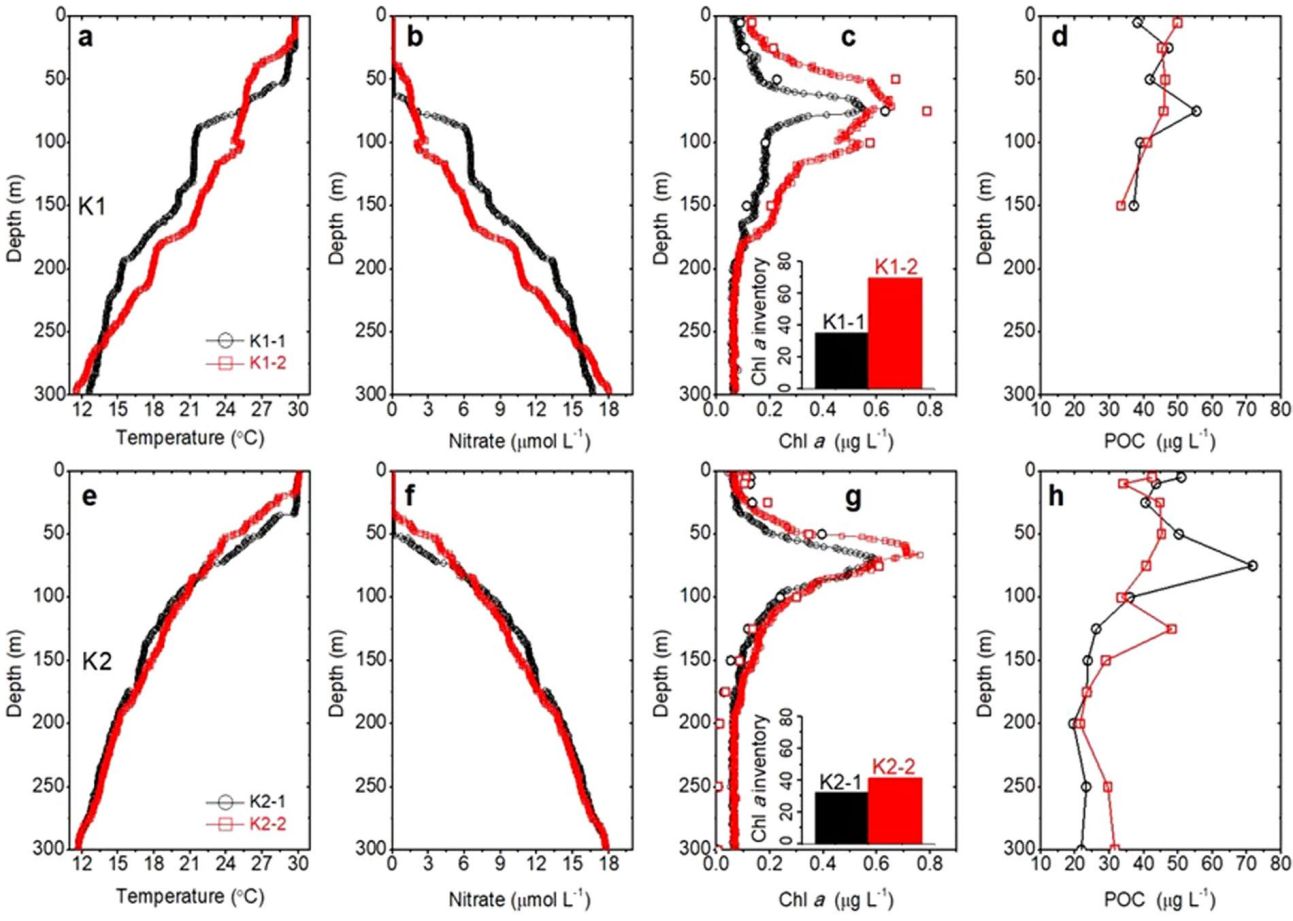
Vertical distributions of temperature (**a**,**e**), nitrate (**b**,**f**), Chl *a* (**c**,**g**), and POC (**d**,**h**) concentrations during different sampling times at stations K1 and K2, respectively. Scatter dots in Fig. 4c,g are Chl *a* values measured by wet chemical method. Bar charts in Fig. 4c,g show integrated Chl *a* inventories of stations K1 and K2, respectively.

Vertical profiles of temperature, nitrate, Chl *a*, and POC concentrations at stations K1 and K2 show conspicuous changes in the euphotic zone (Fig. 4) after the passage of internal waves. Surface nitrate concentrations during or after the internal waves periods were below the detection limit (<0.1 μmol L^−1^) (Fig. 4). However, nitrate inventories integrating from surface to the subsurface Chl *a* maximum depth (0–75 m) were 18.34 and 46.78 mmol m^−2^ (Table 1 and Fig. 4) during and after internal waves at station K1, respectively. Similar case was observed at station K2 with nitrate inventories of 64.97 and 72.60 mmol m^−2^ during and after internal waves, suggesting internal waves may affect nutrient supply in the euphotic zone.

**Table 1:** POC flux and other biogeochemical parameters in the northern South China Sea.

Station		Date (dd/mm/yy)	EZ (m)	In-Nitrate (mmol m^−2^)	In-Chl *a* (mg m^−2^)	In-POC (g m^−2^)	Internal wave influence	POC flux (mg m^−2^ d^−1^)
K1	K1-1	06/08/16	152	18.34	34.86	6.46	Yes	110.9 ± 10.7
	K1-2	06/08/16	142	46.78	69.91	6.45	Yes	
K2	K2-1	04/08/16	132	64.97	32.38	6.53	Yes	54.3 ± 7.7
	K2-2	04/08/16	118	72.60	41.53	6.11	Yes	
H1		Summer^a^	NA^b^	4.87–97.87	31.64–40.09	3.95–5.51	Non	42.0–73.0
H2		05/09/12	NA^b^	3.58	35.39	4.79	Non	32.6 ± 2.7
H3		25/09/12	NA^b^	33.99	31.30	4.90	Non	36.2 ± 3.8

^a^Summer cruises at station H1 were conducted on 24 June 2014 and 25 September 2012. NA^b^ is the abbreviation for not available.

Integrated Chl *a* inventories at stations K1 (34.9 vs. 69.9 mg m^−2^) and K2 (32.4 vs. 41.5 mg m^−2^) also showed significant difference during and after internal waves (Table 1 and Fig. 4c,g). As mentioned above, the elevated Chl *a* at station K1 might be due to the enhanced vertical mixing by internal waves, and transferring cold and nutrient-replete subsurface water to the surface euphotic zone to support biological activity. The enhanced Chl *a* inventory after internal waves may be related to nutrient supply in the euphotic zone. However, it is difficult to explain that phytoplankton can flourish double during a short time period at station K1 (<12 hours). One possibility is that there are many internal waves passing the study area according to SAR images (Fig. 1), and the enhanced Chl *a* may be supported by the enhancement of vertical mixing via earlier internal waves which bring cold and nutrient-replete water into the euphotic zone before the deployment at station K1 on August 6 (Fig. 4), evidenced by the Terra MODIS image that shows internal waves at station K1 on August 4 (Fig. 3). Namely, the enhanced phytoplankton biomass affected by the internal wave packets should have a time lag or due to shipboard location shift. Moreover, injected CDOM from the deep water to the euphotic zone may cause partial contribution to Chl *a* concentration^16^, but it is difficult to estimate CDOM’s influence on Chl *a* in this study. Alternately, phytoplankton transported by internal waves from deep depths or other areas may partly explain Chl *a* inventory changes. Furthermore, the vertical variations of Chl *a* at stations K1 and K2 may be partly caused by the photo-adaptation of phytoplankton assemblages when they experienced varied irradiance at different depths^24,25^.

### Enhanced POC flux and potential mechanisms

POC flux at station K1 was 110.9 ± 10.7 mg m^−2^ d^−1^ (Table 1) which is much larger than previously reported POC flux (32.6–73.0 mg m^−2^ d^−1^) in the NSCS without influence of internal waves and extreme atmospheric events^26–28^ (Table 1). The measured POC flux this study is based on the same method as previous reports of Huang^27^ and Hung *et al*.^26,29^, thus the possible bias in POC export method to our results should be insignificant. Here, we propose several mechanisms to explain elevated POC flux at station K1.

First, in longer time scale, POC export flux was regulated partly by POC stock in euphotic zone^30^, which was strongly related to Chl *a* concentration as they are both originated from biological production. We diagnosed if POC export was affected by biogenic phytoplankton biomass (e.g. Chl *a* values). In the time series record (Fig. 2a), Chl *a* concentration increased from <0.10 to 0.25 μg L^−1^ at ~160 m during warm shock period from 10~12 am on 6^th^ August. Areal Chl *a* stock at K1-2 (69.91 mg m^−2^) after internal waves is two folds of that at K1-1 (34.86 mg m^−2^). As mentioned above, flourished phytoplankton in water column might result from nutrient supply from subsurface, CDOM effect or phytoplankton shift from other areas or depths. According to our measured Chl *a* values by a fluormeter after extraction with acetone, they matched well with CTD-fluorescence-derived Chl *a* values (Fig. 4c,g) so that the effect of CDOM may not be important here. Instead, nutrient-stimulated phytoplankton biomass (i.e. POC) increase may likely play a role contributing some of POC flux. This can be interpreted as follows: internal waves enhance vertical mixing that entrains cold and nutrient-replete water to the upper euphotic zone in the dissipation zone, stimulates phytoplankton growth (the amount of nutrient will be estimated in later section), and then fuels zooplankton and biological activity.

Secondly, one may note that the water in the subsurface layer (80–150 m) at station K1-2 was much warmer than K1-1 (Fig. 4a), consistent with time series temperature graph in which warmer water was carried into deep depths during 10~12 am. As a consequence, phytoplankton may be killed by sudden temperature change either cold shock or warm shock. According to the investigations, phytoplankton cannot survive after sudden temperature change^31^. Therefore, these dead phytoplankton may enhance POC flux partly.

Moreover, to better understand how much nutrient supply influenced by internal waves, we simply use a diffusion model to estimate upward nutrient flux in the NSCS. Generally, moderate POC flux of 32.6–73.0 mg m^−2^ d^−1^ are reported in the oligotrophic water of NSCS in summer (June and September) under non-typhoon conditions^26–28,30^, it means it needs new nitrogen (in dissolved inorganic form) of 298–912 μmol N m^−2^ d^−1^ if a C/N ratio of 6.6 is applied^21^. The sources of dissolved inorganic nitrogen in the water column could come from atmospheric (including dry and wet) deposition, phytoplankton fixation, and diffusion from subsurface. The amount of atmospheric deposition and phytoplankton fixation approximately account for 56~204 μmol m^−2^ d^−1^ (Table 2), on average 130 μmol m^−2^ d^−2^, which is much lower than new nitrogen value needed (298–912 μmol m^−2^ d^−1^) (Table 2). If we use nitrate gradient of 0.06 mmol m^−4^ below mixed layer^27^, diffusivity rate of 4.9 × 10^−4^ m^2^ s^−1^ during internal waves condition^11^, the upward diffusion flux could be 2540 (0.06 mmol m^−4^ × 4.9 × 10^−4^ m^2^ s^−2^ × 86400 × 1000) μmol N m^−2^ d^−1^ triggered by internal waves. Using this estimated flux we next assume that the effective diffusion time is 6 hours (Fig. 2a) during the dissipation zone affected by internal waves, the daily nitrate diffusion flux will be 635 μmol N m^−2^ d^−1^ (Table 2). Nitrate diffusive flux triggered by internal waves is much larger than the maximum nitrogen fixation^32,33^, and atmospheric deposition reported^34–36^ (Table 2), supporting a new production of 50.3 mg C m^−2^ d^−1^. Overall, our filed observation shows that enhanced vertical mixing induced by internal waves transport considerable nutrients into the upper euphotic zone, and stimulate phytoplankton growth and other biological activity. But POC export flux conveyed by sinking particles, collected by sediment traps, may have a time lag from phytoplankton peak since it is difficult to understand how many internal waves passed the study area before our field cruise.

**Table 2:** Nutrient supplies during internal waves condition in the northern South China Sea.

Nitrogen flux^a^ (μmol m^−2^ d^−1^)	POC flux^b^ (mg m^−2^ d^−1^)	Position	Period	Method
**Diffusion**				
635	50.3	NSCS	Summer	This study
**Phytoplankton N**_**2**_ **fixation**				
12.6 ± 5.7	1.0 ± 0.4	20–21.5°N,118–120°E	June-August	^15^N incubation^32^
57.5 ± 71.2	4.6 ± 5.6	SEATS^c^	August	Mass balance model^33^
**Dry deposition**				
13.7 ± 5.5	1.1 ± 0.4	EANET^d^ remote sites	Four seasons	Deposition sampling^34^
62.0 ± 35.0	4.9 ± 2.8	Dongsha Atoll	July	Deposition sampling^35^
**Wet deposition**				
30.0 ± 10.0	2.4 ± 0.8	SCS	Summer	Model^21^
84.9	6.7	Yongxing Island	Summer	Rainwater sampling^36^

Nitrogen flux^a^ was calculated based on nitrogen supply in dissolved inorganic forms. POC flux^b^ was calculated based on nitrogen flux and C/N ration of 6.6. SEATS^c^ is the abbreviation for South East Asia Time-Series Station. EANET^d^ is the abbreviation for Acid Deposition Monitoring in East Asia.

Finally, the POC flux of station K2 was 54.3 ± 7.7 mg m^−2^ d^−1^ which is much less than the flux of station K1 but is close to POC flux (32.6–73.0 mg m^−2^ d^−1^) at stations H1, H2 and H3 during non-typhoon conditions in summer. We also observed strong influence of internal waves which transport POC downward at station K2 (Fig. 4h). There were also Chl *a* increase in K2-2 (Fig. 4g), indicated possibly biological production. Less POC export at station K2 may be explained by the sake of the transmission zone influenced by internal waves since the entrained nutrient at station K2 is much lower than station K1. Alternatively, it may be caused by tilting of sediment traps during rapid rise of trap (~32 m within 10 min), but it is difficult to quantify possible trapping efficiency when traps are tilt.

## Materials and Methods

Sinking particles were collected at ~150 m (below euphotic zone) using a drifting sediment trap array^29,37^. The trap deployment periods were 14 hours atstationK1 (6^th^ August), and 24 hours at station K2 on (4^th^ August), respectively (Fig. 1). Swimmers evident with a microscope on the filters were carefully removed using forceps. Attached to the trap, a conductivity-temperature-depth sensor (CTD, Ocean Seven 310 Multi parameter Probe) was deployed at stations K1 (~160 m) and K2 (~180 m) to record the hydrological properties of water column, respectively.

In addition, we collected seawater samples from water column twice for each trap station aboard RV OR-3. Here, K1-1 and K1-2 represent sampling time at 9 am and 5 pm on 4^th^ August at station K1, respectively. K2-1 and K2-2 represent sampling time at 5 am and 11 am on 6^th^ August at station K2, respectively. A SBE9/11 plus CTD sensor was used to measure hydrographic conditions. Concentration of POC in suspended and sinking (trap collected) samples were measured using an elemental analyzer (Elementa, Vario EL-III, Germany), according to Hung *et al*.^26^. Concentration of Chl *a* was measured with a Turner Designs 10-AU-005 fluormeter after extraction with 90% acetone using non-acidification method^37^. Nitrate concentrations at stations H1, H2 and H3 were measured according to Gong *et al*.^38^. Concentrations of nitrate at stations K1 and K2 were calculated using linear regression result between nitrate and temperature^39^. Integrated nitrate (In-Nitrate, 0–75 m), Chl *a* (In-Chl *a*, 0–150 m), and POC (In-POC, 0–150 m) stocks were calculated using trapezoidal rule. A PAR scalar quantum irradiance sensor (Chelsea Technologies Group Ltd, UK) was used to calculate the euphotic depth (EZ) which was defined as the depth of 0.1% surface light penetration.
